# Differences between recreational gamers and Internet Gaming Disorder candidates in a sample of *Animal Crossing: New Horizons* players

**DOI:** 10.1038/s41598-023-32113-6

**Published:** 2023-03-29

**Authors:** Moritz Wischert-Zielke, Antonia Barke

**Affiliations:** 1 Department of Clinical Psychology and Department of American Studies, Catholic University of Eichstätt-Ingolstadt, Eichstätt, Germany; 2 Clinical Psychology and Psychological Interventions, Institute of Psychology, University Duisburg-Essen, Essen, Germany

**Keywords:** Psychology, Diseases, Risk factors

## Abstract

Throughout the last decade, research has considered players’ gaming motives as risk and the perceived social support (PSS) as protective factors in the context of Internet Gaming Disorder (IGD). However, the literature is lacking diversity regarding the representation of female gamers as well as of casual and console-based games. The aim of this study was to assess IGD, gaming motives, and PSS comparing recreational gamers and IGD candidates in a sample of *Animal Crossing: New Horizons* players. A total of 2909 ACNH players (93.7% of them female gamers) took part in an online survey which collected demographic, gaming-related, motivational, and psychopathologic data. Using the cut-off of at least five positive answers to the IGDQ, potential IGD candidates were identified. ACNH players reported a high prevalence rate for IGD (10.3%). IGD candidates differed from recreational players regarding age, sex, and game-related, motivational, and psychopathological variables. A binary logistic regression model was computed to predict membership in the potential IGD group. Age, PSS, escapism and competition motives as well as psychopathology were significant predictors. To discuss IGD in the context of casual gaming, we consider demographic, motivational, and psychopathological player characteristics as well as game design and the COVID-19 pandemic. IGD research needs to broaden its focus concerning game types as well as gamer populations.

## Introduction

Video gaming, for most people across all age groups, is an entertaining leisure activity which may support mental health and overall well-being^1^. Particularly as a social experience, video games provide digital places for social interactions in which gamers may express themselves in novel ways and create emotional relationships^2–5^.

For a small portion of gamers, however, gaming can become a problematic experience resulting in marked distress or impairment of personal or social functioning^6^. Hence, the *Diagnostic and Statistical Manual of Mental Disorders* (DSM-5) included Internet Gaming Disorder (IGD) as a psychiatric disorder candidate in 2013 paving the way for a more standardized line of clinical research^7^. With the endorsement of the 11th version of the International Classification of Diseases and Related Health Problems (ICD-11) by the World Health Organization in 2019 Gaming Disorder became a second conceptualization of the clinical phenomenon of problematic gaming^8^. While the DSM-5 includes nine criteria (i.e., preoccupation; withdrawal; tolerance; unsuccessful attempt to control gaming behavior; loss of interest in previous hobbies; continued excessive use of internet games despite knowledge of psychosocial problems; deceiving others regarding the amount of gaming; playing games to escape a negative mood; risking or losing significant relationships or opportunities because of playing), the ICD-11 lists three core criteria (impaired control over gaming; increasing priority given to gaming to the extent that gaming takes precedence over other life interests and daily activities; and continuation or escalation of gaming despite the occurrence of negative consequences). As neither the two official conceptualizations nor scholars in the field agree on a single set of diagnostic criteria, further research is direly needed before a unitary framework can and should be accepted in the field of research^9^.

Recent meta-analyses report worldwide prevalence rates of IGD between 2.5 and 4.6%^10–13^, although individual studies may vary widely^14^. Literature suggests close associations between IGD and psychological problems in general^15^, and depression^16–22^, anxiety^16,18,19,23,24^ and ADHD^16,19,25–29^, in particular.

Since mere time spent on gaming was found to be a poor predictor of negative health outcomes of players and IGD^30–34^, research has explored associated risk and protective factors. IGD appeared associated with younger age^10,35–37^ and male sex^11,13,16,19,21,36,38^.

One branch of research has focused on gaming motives as the DSM-5 criteria include the motivation to play “to escape or relieve a negative mood” (p. 795)^7^. In their attempt to merge previous theoretical models of IGD, Young and Brand regard gaming motives as part of a person’s “core characteristics and [. . .] therefore important predictors of the development and maintenance of IGD” (p. 6)^39^. Following pioneering work by Bartle^40^ and Yee^41^, motivations related to social, achievement (or skill-related), and escapist elements recurred in most factor analyses^41–47^. The widely used *Motives for Online Gaming Questionnaire* (MOGQ) by Demetrovics and colleagues^48^ distinguishes seven independent motivational factors: *social*, *escape*, *competition*, *coping*, *skill development*, *fantasy*, and *recreation*. Evidence mounts that gaming motives are important predictors of IGD (see^49–54^). A recent meta-analysis on risk and protective factors by Ropovik and colleagues^21^ reported pooled associations of the seven motives to IGD between 0.08 and 0.42 (escape *r* = 0.42, fantasy *r* = 0.32, coping *r* = 0.30, competition *r* = 0.24, skill development *r* = 0.21, social *r* = 0.19, recreation *r* = 0.08). A second meta-analysis replicated the strongest association of IGD with the escapism motivation^55^, whereas a third found the escapist association to be slightly surpassed by achievement motivation in the Chinese population^36^.

On the side of potential protective factors in the context of IGD some pioneering studies have explored social support, and—to emphasize particularly its subjective perception—*perceived* social support (PSS), as an indicator of a person’s perception of available interpersonal resources (see^56–60^). Two recent meta-analyses reported small associations of IGD and PSS of *r* = -0.05^21^ and *r* = − 0.16^36^. However, Teng and colleagues^61^ used a longitudinal design and showed that IGD negatively affects PSS but not vice versa—a finding replicated by Tham and colleagues^62^. In accordance with this, two studies that tested the effect of PSS as a direct predictor on IGD yielded no significant results^57,60^. In sum, the literature offers some evidence that the PSS is negatively associated with IGD, but none supporting its role as a protective factor in a strictly causal sense. As (lack of) social support could play a role in the development and maintenance of IGD, further investigation is called for. Particularly in life-simulation games which thematize social themes, the role of PSS needs further investigation as a lack of PSS might be relevant for gaming motives.

Besides gaming motives and the PSS, the present study aimed to address three issues in particular which require critical attention and further research.

First, there is a need to account for diversity regarding game types and gaming populations. Many studies have focused on so-called Massively-Multiplayer-Online-Role-Playing-Games (MMORPGs) and their player-bases; only recently a more thorough recognition of the variability of games has begun: IGD-scores^63,64^ as well as gaming motives^65,66^ and their prediction values for IGD^67^ vary with the games (and populations) studied. In their meta-analysis, Ropovik and colleagues^21^ explicitly called for an “improved reporting” (p. 16) of game genre. Here, particularly casual games deserve further attention.

Second, and beyond the notions of genre, research has only begun to overcome its initial focus on PC-based games and consider other gaming platforms and the associated patterns in gaming motives and IGD prevalence. Only recently have researchers turned to assessing problematic mobile gaming which, e.g., uses smartphones and tablets rather than PC^68^.

Thirdly, the role of sex/gender remains a crucial issue. Male sex has clearly emerged as a statistically distinct risk factor for IGD. However, research has not yet fully come to terms with the popular “male gamer stereotype, which may negatively reflect on female gamers, who are not yet considered ‘real’ or ‘hardcore’ gamers” (p. 2)^69^. In their effort to conceptualize a female gaming profile, Lopez-Fernandez and colleagues^69^ found a relatively small prevalence of 1% for IGD in their (all-female) sample. McLean and Griffiths argue that “[m]uch of the previous research on online gaming has predominantly used male participants” (p. 971)^70^. Indeed, female gamers are still heavily underrepresented in studies on IGD with a ratio of only 21% in a recent meta-analysis by Ropovik and colleagues^21^ and 30.1% in a systematic review by Şalvarlı and Griffiths^71^.

The present study aimed to expand current research on IGD and the associated factors of gaming motives and PSS. To shift the focus towards (a) casual games, (b) alternative gaming platforms, and (c) female gamers, we chose to focus solely on the recent mobile and life-simulation game *Animal Crossing: New Horizons* (ACNH) for the Nintendo Switch. ACNH has been extremely popular since its release in 2020 with more than 37 million copies sold worldwide^72^. In the game, players can casually build, decorate, dwell in, and share their personal island places with anthropomorphic NPCs (non-player-characters) or other players.

Though there is no universal definition of what people describe as a “casual game,” Kuittinen and colleagues^73^ have discussed and integrated various conceptualizations of “casual gaming”, “casual games”, “casual players”, etc. Following the definition provided by the authors (p. 107), we would describe ACNH as a “casual game” in that (1) its island utopia theme appeals to a wide audience, (2) as a (mobile) console game it is accessible via easy controls, (3) one can learn its gameplay quickly and without much effort, (4) it does not put pressure on the player-character while still constantly offering small rewards for playing (farming, daily rewards like changing items in the shop, in-game mail etc.), and (5) it supports shorter play sessions to players who aim to just briefly check on what is new with their island.

A previous study focusing on ACNH reported a large ratio of female to male gamers in its sample^74^. To the knowledge of the authors, the ACNH community has not been examined with regard to IGD.

We formulated the following hypotheses: Among the adult population of ACNH players, more men rather than more women report IGD (H1.1), IGD candidates are younger (H1.2), play more often (H1.3) and longer per session (H1.4), spend more time on ACNH-related (H1.5) as well as other media (H1.6), spend more time playing other games (H1.7), and spend more money on ACNH content (H1.8) compared to recreational gamers. Hypotheses H.1.2. to H.1.8. thus reflect the preoccupation/increased priority criteria which we expect to result in greater amounts of time spent not only on gaming but also related activities. In view of the literature discussed above, we furthermore hypothesized that IGD candidates report stronger gaming motives (H2.1–7), lower PSS (H3.1) and higher psychopathology scores with regard to somatization, depressive, and anxious symptoms as well as overall psychopathology (H3.2–5) compared with recreational gamers.

## Methods

### Participants and procedure

An online survey was implemented via Qualtrics^75^ and the link distributed to English-speaking ACNH players via Twitter, online forums, and Facebook via posting a short description of the survey. Data collection was conducted between November 2021 and February 2022. No incentives were offered. Before starting the online survey, all participants were informed about the goals of the survey. They were only able to access the survey after providing informed consent. Inclusion criteria were: at least 18 years of age and not under the influence of drugs.

**Figure 1:**
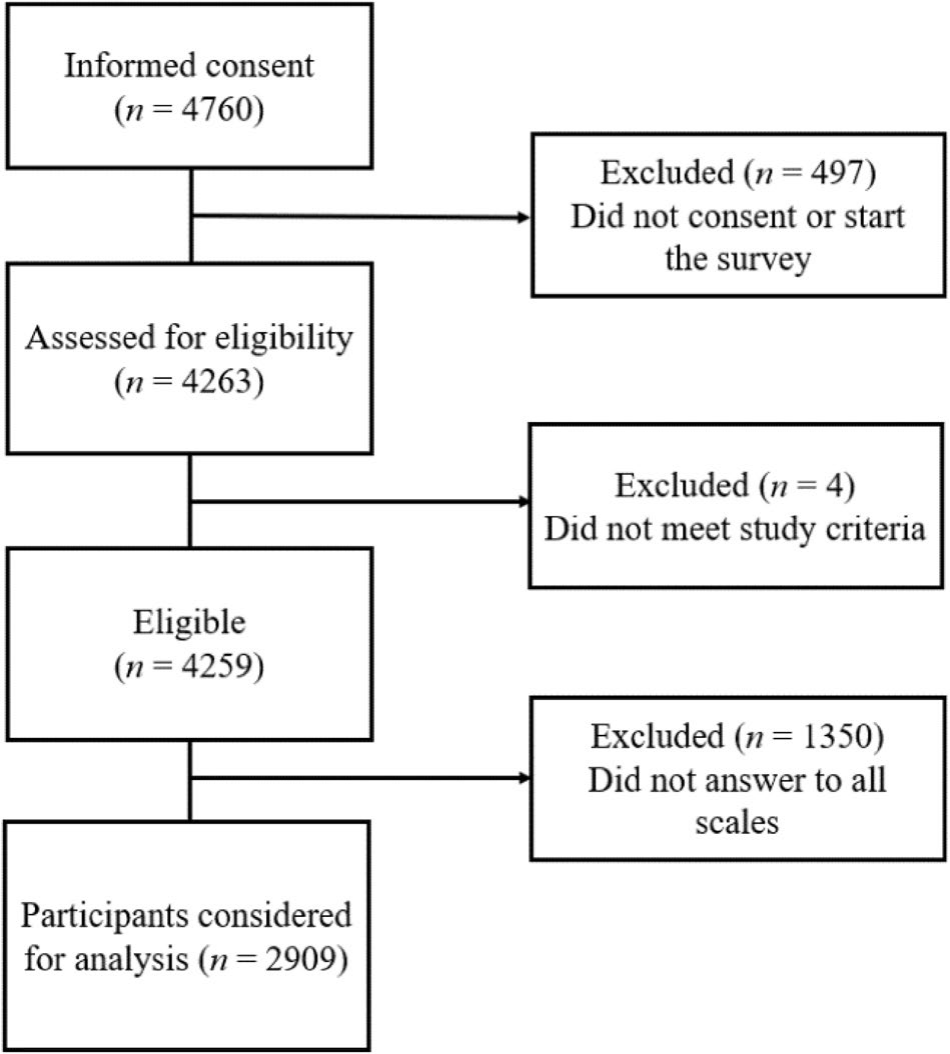
CONSORT flowchart of participants of the present study.

A self-selected sample of 4760 players initially followed the link (see Fig. 1). After excluding participants who did not meet the inclusion criteria or did not answer all scales needed for the statistical analysis, a final sample of N = 2909 participants remained.

Data of N = 2909 participants (women: 2725; men: 135; non-binary: 49) with a mean age of 31.1 ± 8.9 years (MD ± SD in the following) were included in the analyses. The participants resided in 52 countries with the majority living in the US (65.7%), Australia (8.5%), UK (8.3%), Canada (6.0%) and Germany (3.4%).

Nearly two thirds (62.6%) of participants reported playing ACNH every day or every second day. Individual gaming sessions lasted 93 ± 74 min with a total gaming time per week (ACNH plus other games) of 15.1 ± 15.3 h. For further sample characteristics, see Table 1 and Table S1.

**Table 1:** Demographic and media-related variables of study sample.

		*n*	*%*
Sex	Female	2725	93.7
	Male	135	4.6
	Non-binary	45	1.5
	Other	4	0.1

	*M*	*SD*	
Age	31.08	8.87	
Gaming time (h/week)	15.06	15.30	
Gaming session duration (min)	92.80	74.20	
Time spent on other games (h/week)	8.98	13.54	
Media use (h/week)	21.13	20.36	
ACNH-related media use (h/week)	6.43	12.38	

Total sample size was N = 2909.

### Measures

Questions relating to general socio-demographic information (e.g.: age, sex, country of residence) and general variables concerning gaming behavior (e.g.: weekly time played, game-related and general media use, weekly time played other games) were collected.

Participants’ motivations for gaming were assessed using the *Motives for Online Gaming Questionnaire* (MOGQ)^48^. The MOGQ is a 27-item self-report measure using a 5-point Likert scale (with 1 = never to 5 = almost always/always). Participants are asked to indicate to which extent they relate their gaming behavior to the following motives: social (e.g.:“…because I can meet many different people”), escape (e.g.: “…to forget about unpleasant things or offenses”), competition (e.g.: “…because it is good to feel that I am better than others”), skill development (e.g.: “…because it improves my skills”), coping (e.g.: “…because it helps me get rid of stress”), fantasy (e.g.: “…to be somebody else for a while”), and recreation (e.g.: “…because it is entertaining”). The MOGQ’s internal structure of seven subdimensions has been confirmed via exploratory factor analysis^76,77^. The scale has demonstrated satisfying internal consistency with Bányai and colleagues^78^ reporting Cronbach's alphas between 0.78 and 0.91 in their sample of N = 4284 gamers, reflected by our own data (Cronbach’s alphas between 0.73 and 0.89 except for the recreation scale at 0.59).

Participants were then asked to relate their PSS using the English version of the *F-SozU K-6* developed by Kliem and colleagues^79^. This brief form is based on the *Fragebogen zur Sozialen Unterstützung*, FSozU, by Fydrich and colleagues^80^ which is comprised of the scales “emotional support,” “practical support,” “social integration,” and “strain from the social network”. The original questionnaire was created in German, but a subsequent cross-cultural validation study with over 30.000 participants across the US, Russia, China, and Germany confirmed its good reliability (with a Cronbach’s alpha of 0.89 for the English version) and strong model fit^81^. The short version^79^ is comprised of six items, which are rated on a 5-point Likert scale from (1 = ”not true at all” to 5 = ”very true”).

To account for problematic gaming, the *Internet Gaming Disorder Questionnaire*, IGDQ^6^ was administered. The IGDQ is a screening instrument based on the nine DSM-5-criteria for IGD. It consists of one dichotomous item (“yes” or “no”) for every criterion and was validated by Jeromin and colleagues^82^. Since the IGDQ is a screening instrument and a clinical diagnosis requires a full diagnostic process conducted by a clinician, we refer to the participants above the cut-off score of 5 as “candidates for IGD”.

Finally, psychiatric distress operationalized through depressive, anxious, and somatoform syndromes was measured using the short form of the *Brief Symptom Inventory* (BSI-18)^83^. The BSI-18 asks participants to rate to what extent they have suffered from the indicated symptoms in the last week on a 5-point Likert scale (from 1 = not at all to 5 = very much). It is a short, reliable instrument for the assessment of psychological distress^84^.

### Statistical analysis

Participants who agreed with 5 or more items of the IGDQ self-rating checklist of DSM-5 criteria for IGD were classified as candidates for Internet Gaming Disorder (IGD_cand_) and formed one group, participants who endorsed fewer items on the IGDQ were classed as recreational gamers (RG). The groups were compared for sex with χ^2^ tests and metric measures (age, total ACNH total gaming time, weekly ACNH gaming time, duration of individual ACNH sessions, additional weekly ACNH-related media time, money spent on game-related purchases and psychometric measures) were compared with t-tests for independent samples. In cases in which the Levene test showed variance inhomogeneity, results of the Welch test were reported and degrees of freedom adjusted accordingly. For the comparison of single-item ordinal ratings of frequency of the in-game activities, Mann–Whitney U tests were used. As measures of effect size, Hedges’ g is reported. To control for multiple comparisons and the presence of the other variables in the calculation, a binary logistic regression with the criterion IGD_cand_ /RG and the predictors age, sex, PSS, gaming motivation and psychopathology was calculated. The method was ENTER and the predictors were entered blockwise in 4 blocks: Block 1: age and sex, Block 2: PSS, Block 3: MOGQ subscales, Block 4: BSI-18 (GSI).

### Ethics

The Ethics Committee of the Catholic University of Eichstätt-Ingolstadt assessed and approved the study protocol on October 10th in 2021. The study procedures were carried out in accordance with the Declaration of Helsinki. All participants were informed about the study and provided informed consent.

### Informed consent

All procedures followed were in accordance with the ethical standards of the responsible committee on human experimentation (institutional and national) and with the Helsinki Declaration of 1975, as revised in 2000. Informed consent was obtained from all patients for being included in the study.

## Results

According to the IGDQ, 301 participants (10.3%) agreed to five or more of the statements, thus making them candidates for IGD according to the DSM-5 criteria. A higher percentage of men reported IGD_cand_ 15.6% (21/135) than women 10.0% (273/2725), χ^2^ = 4.28, *p* = 0.043. Participants with IGD_cand_ (28.6 ± 7.1) were younger than their counterparts (31.4 ± 9.0) [Welch test: *t*(415.024) = 6.11, *p* < 0.001, *d* = 0.31]. Accordingly, hypotheses H1.1. and H1.2. were confirmed.

The IGD group reported longer weekly gaming times, longer individual gaming sessions and more ACNH-related additional media time and spent more real money on additional game-related content (see Table 2 for details). The IGD group was also more likely to have restarted their island (χ^2^ = 8.40, *p* < 0.01), but did not differ with the frequency of other in-game activities except that they reported more frequent interactions with strangers (Table 3) than the recreational gamers. Therefore, hypotheses H1.3.-8 were confirmed.

**Table 2:** Group differences regarding age, gaming time, and money spent on game-related purchases.

Variable	IGD candidates		Recreational gamers		*t*	*df*	*p*	*g*
	M	SD	M	SD				
Age	28.6	7.1	31.4	9.0	6.11	415.024^a^	< 0.001	0.31
ACNH gaming time (h/week)	22.9	20.1	14.2	14.4	− 7.35	334.144^a^	< 0.001	− 0.58
Session duration (min)	116.1	79.8	90.1	73.1	− 5.36	354.232^a^	< 0.001	− 0.35
ACNH-related media time (h/week)	10.7	18.6	6.0	11.4	− 4.25	314.001^a^	< 0.001	− 0.39
Time spent on other games (h/week)	14.9	18.7	8.30	12.7	5.9	323.239^a^	< 0.001	− 0.49
Total recreational online time (h/week)	26.2	22.8	20.6	20.0	4.06	341.747^a^	< 0.001	− 0.28
Real money spent on game (US dollars)	52.3	159.5	30.8	131.0	− 2.25	347.787^a^	0.013	− 0.16

^a^Welch’s test is reported due to variance inhomogeneity and the degrees of freedom corrected accordingly.

**Table 3:** Group differences in in-game activities.

Variable	IGD candidates		Recreational gamers		*U(df* = *1)*	*p*	*g*
	M	SD	M	SD			
Interaction with friends	2.6	1.0	2.6	1.0	388,538.0	0.798	− 0.01
Interaction with strangers	2.5	1.0	2.4	0.9	422,452.5	0.018	− 0.16
Interaction with NPCs	4.3	0.8	4.3	0.8	391,986.5	0.947	− 0.01
Visiting others’ islands	2.7	1.0	2.7	0.90	382,523.5	0.450	− 0.04
Receiving visitors on one’s own island	2.5	0.9	2.5	0.8	382,399.0	0.524	0.01
Trading activities	2.7	1.1	2.6	1.0	409,275.0	0.151	− 0.11
Sending letters or gifts	2.4	1.0	2.5	1.1	372,833.0	0.194	0.08

**Figure 2:**
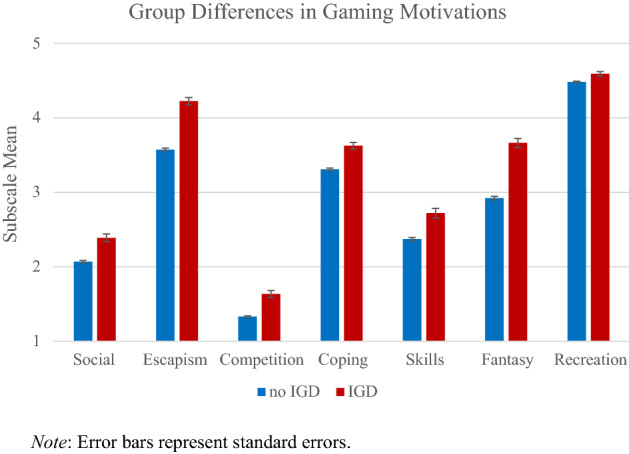
Gaming motivations as a function of group membership (recreational users vs. candidates for IGD)**.** Error bars represent standard errors.

Participants of the IGD group reported higher scores for all gaming motivation subscales (Fig. 2, Table 4) with the largest differences for the subscales fantasy (*g* = 0.65), escape (*g* = 0.59) and competition (*g* = 0.55). Hypotheses H2.1–7 were confirmed.

**Table 4:** Group differences in gaming motivations (MOGQ) as well as in psychopathological symptoms (BSI-18).

Variable	IGD candidates		Recreational gamers		*t*	*df*	*p*	*g*
	M	SD	M	SD				
Social	2.4	0.9	2.1	2.4	− 6.26	2904	< 0.001	− 0.38
Escapism	4.2	0.8	3.6	4.2	− 12.24	433.848^a^	< 0.001	− 0.59
Competition	1.6	0.8	1.3	1.6	− 6.47	330.408^a^	< 0.001	− 0.55
Coping	3.6	0.7	3.3	3.6	− 6.64	2905	< 0.001	− 0.40
Skills	2.7	1.1	2.4	2.7	− 5.25	2904	< 0.001	− 0.32
Fantasy	3.7	1.0	2.9	3.7	− 11.58	389.995^a^	< 0.001	− 0.65
Recreation	4.6	0.5	4.5	4.6	− 3.29	390.420^a^	< 0.001	− 0.18
BSI somatization	7.3	5.5	4.5	4.7	− 8.55	352.069^a^	< 0.001	− 0.59
BSI depressive symptoms	12.6	6.7	7.9	6.3	− 11.47	2904	< 0.001	− 0.70
BSI anxiety symptoms	10.7	6.8	6.9	5.8	− 9.34	351.189^a^	< 0.001	− 0.65
BSI GENERAL symptom index	30.3	16.5	19.3	14.7	− 11.13	357.311^a^	< 0.001	− 0.74

*BSI* Brief Symptom Index (Ref).

^a^Welch’s test is reported due to variance inhomogeneity and the degrees of freedom corrected accordingly.

The participants of the IGD group reported less PSS (21.4 ± 5.9) than those in the recreational gamer group (23.4 ± 5.6) [Welch test: *t*(364,314) = 5.82, *p* < 0.001, *g* = 0.37) and more psychopathology than those in the non-IGD group (0.54 < *g* < 0.74) (see Table 4 for details). Thus, the final hypotheses H3.1–5 were confirmed.

To integrate these results with each other and account for the presence of the variables in a general model, we calculated a binary logistic regression with the criterion IGD_cand_ / RG. The regression showed that younger age, escapist and competitive gaming motivation, a perceived lack of social support and general psychopathology contributed to the group membership of the IGD group, with sex narrowly losing its significance once the gaming motivations were entered (see Table 5).

**Table 5:** Binary logistic regression with the criterion IGD_cand_/RG.

	Wald (*df* = 1)	*p*	OR	OR 95% CI Lower	OR 95% CI Upper	∆R^2^	χ^2^	*df*	*p*
Model 1^a^						0.023	6.22	2	< 0.001
Constant	1.18	0.277	0.683						
Age	24.27	< 0.001	0.958	0.942	0.974				
Sex^b^	4.49	0.034	0.591	0.364	0.961				
Model 2						0.024	32.70	3	< 0.001
Constant	4.86	0.027	2.50						
Age	23.74	< 0.001	0.958	0.942	0.975				
Sex^b^	4.02	0.045	0.606	0.372	0.989				
PSS	34.22	< 0.001	0.942	0.924	0.961				
Model 3						0.090	129.92	7	< 0.001
Constant	15.07	< 0.001	0.061						
Age	11.46	0.001	0.969	0.952	0.987				
Sex^b^	3.32	0.069	0.621	0.371	1.037				
PSS	16.92	< 0.001	0.956	0.936	0.977				
MOGQ social	0.00	0.986	1.000	0.958	1.044				
MOGQ escapism	19.94	< 0.001	1.127	1.070	1.188				
MOGQ competition	32.29	< 0.001	1.150	1.096	1.207				
MOGQ coping	2.29	0.130	0.951	0.891	1.015				
MOGQ skills	0.02	0.899	0.998	0.962	1.035				
MOGQ fantasy	6.91	0.009	1.054	1.013	1.096				
MOGQ recreation	0.36	0.551	1.027	0.941	1.120				
Model 4						0.020	30.22	1	< 0.001
Constant	24.31	< 0.001	0.026						
Age	5.14	0.023	0.979	0.961	0.997				
Sex^b^	3.81	0.051	0.596	0.355	1.002				
PSS	4.78	0.029	0.975	0.953	0.997				
MOGQ social	0.04	0.834	1.005	0.962	1.049				
MOGQ escapism	11.48	0.001	1.097	1.040	1.157				
MOGQ competition	33.00	< 0.001	1.154	1.099	1.212				
MOGQ coping	2.92	0.088	0.945	0.885	1.008				
MOGQ skills	0.01	0.945	0.999	0.962	1.036				
MOGQ fantasy	3.54	0.060	1.039	0.998	1.081				
MOGQ recreation	0.73	0.393	1.039	0.952	1.134				
BSI-18 (GSI)	30.45	< 0.001	1.026	1.016	1.035				

^a^χ^2^ values refer to the increments compared to the previous restricted models. The values for the whole models are as follows: Model 2: χ^2^ = 64.97, *df* = 3, *p* < 0.001, Nagelkerke’s R^2^ = 0.047; Model 3: χ^2^ = 194.89, *df* = 10, *p* < 0.001, Nagelkerke’s R^2^ = .137; Model 4: χ^2^ = 225.11, *df* = 11, *p* < 0.001, Nagelkerke’s R^2^ = .157; Hosmer–Lemeshow-Test for final model *p* > 0.20.

^b^Reference Category: Men.

## Discussion

The present study examined IGD, psychopathology, the gaming motives, and the PSS in a sample of ACNH-players for the first time. We thus focused solely on a “casual” and console-based game and its player base. Surprisingly, however, we found an overall prevalence rate of potential IGD of 10.3% in our sample, which is twice the rates reported by current meta-analyses^10–13^. Additionally, this finding contrasts with a recent study in which the use of PC but not of other consoles was a significant predictor of IGD^85^. Our results add to the literature on console and casual games which are still underrepresented in research on IGD.

Moreover, our findings contribute to a better representation of female gamers in IGD research. Although our sample consisted mostly of women, the ratio of IGD candidates to recreational gamers was still higher in male than in female gamers (10% for female gamers, 15.6% for male gamers). This is in line with findings that male sex is a risk factor for IGD^11,13,16,19,21,36,38^ and not just an artefact of the preponderance of male gamers in the games most studied. Even in the context of a game mainly played by women, male sex was still associated with excessive gaming. This finding is, however, complicated by the unequal distribution of male and female gamers in our sample and should be received with caution. Regarding the predictive potential in our regression model, sex did no longer yield a significant result when considered together with the other factors (age, gaming motives, PSS, and psychopathology). Understanding sex differences in IGD and its treatment^86^ may be advanced by studying underlying factors in female and male gamers.

As to the question why gaming can become problematic for some gamers but not for others, our results suggest that among ACNH players, IGD candidates differ from recreational players in demographic, game-related, and motivational aspects, as well as psychopathology and PSS. First, IGD candidates were younger, played more hours per week and their gaming sessions lasted longer. They spent more time on ACNH-related as well as other media and spent more money on game-related content than recreational players. Strikingly, the two groups did not differ regarding particular in-game activities such as interaction with NPCs and other players, or trading. This indicates that those playing in a problematic way do not play the game itself differently—although they may be differently motivated.

Secondly, IGD candidates indicated higher gaming motives for all subcomponents of the MOGQ, the motives “escapism”, “fantasy”, and “competition” showed the largest effect sizes in the comparisons with recreational gamers. Many previous studies have shown strong associations of IGD to escapism^34,85,87–95^ and fantasy^76,85,88,91–94^ but also the coping motive^88,91,92,94,96,97^. Regarding escapist and coping motives, which are as well part of the nine IGD criteria, future research needs to scrutinize their relevance for alternative conceptualizations of gaming disorder (particularly those of the ICD-11^8^) which do not already overlap with the constructs.

While escapism was indeed a significant predictor in the regression model, coping was not. Here, our findings on the ACNH community differ from previous studies^52,87,91,96^ which have made a strong case for gaming as a coping strategy. Furthermore, our results draw attention to the competition motive of IGD candidates, which was the second significant predictor in our regression model. Whereas studies have reported close associations of IGD to competition motivation^88,89,92^, this appears in conflict with the image of ACNH as a “casual” game and the game mechanics as they are marketed and perceived. For ACNH and the related franchise, game mechanics and story explicitly avoid competitive themes and in-game practices among players (unlike, e.g., shooters and MMORPGs). However, possibly competitive dynamics may evolve in playing practices within and between players regarding aesthetic or resource-related elements or time-consuming unlocking of specific items or characters. This may further be facilitated by affect-driven social media platforms like Facebook and YouTube which circulate content on ACNH and the overall endeavor of creating an island as unique and characteristic as possible. The fact that IGD candidates were more likely to restart the game over from scratch and to reshape their island may be read as an indicator of this valued but hard-to-realize goal.

Thirdly, ACNH players who screened positively for IGD showed higher overall psychopathology, as indicated by BSI-18 scores. Particularly the depression and anxiety subscales differed between potential IGD candidates and recreational gamers whereas all three dimensions showed robust effect sizes. In addition, the general BSI-18 score was as well a significant predictor of IGD in the regression model. Both findings add to the growing relevance of IGD as a phenomenon of clinical and social relevance.

Finally, IGD candidates reported lower levels of PSS compared to recreational gamers. In line with previous studies, PSS was associated with IGD, although due to the design no causal conclusions may be drawn. Nevertheless, as PSS was as well a significant predictor of IGD in our regression model, our results add to the literature that (lack of) social support may be a vital factor in the prediction of IGD and should be taken into account in prevention and psychotherapy. As beyond this, neither coping nor social motivations for gaming were significant predictors of IGD-scores in our sample, our results do not allow to conclude that problematic gaming is to be thought merely as a mal-adaptive attempt at compensating for a lack of social support. Here longitudinal designs should be used in future studies to examine the causal relations between social support and IGD, as well as their ties to gaming motives.

Beyond the variables assessed in our study, the ongoing consequences and social and cultural changes coming with the COVID-19 pandemic must be considered in IGD research^98^. First studies have found evidence for increased IGD prevalence rates with the onset of the pandemic^99,100^. Further research is needed to assess the nature and stability of possibly heightened prevalence rates particularly comparing multiple measures of problematic gaming.

Further factors for why ACNH might lead some players to IGD may be the open-ended nature of the gameplay together with elements that reward players for playing daily. In this context, specific game mechanics such as reward contingencies and the way in which they influence and shape players’ cognitive and emotional responses should be examined^101^. Recent studies have, for example, already begun to take into account the role of fear of missing out in IGD (see:^102–105^) or the randomization of unlockable content^106^. Here, it might be fruitful to study the interaction of game design features with player characteristics such as reward-related deficits in decision-making^107^.

With the substantial sample size and a large percentage of female gamers this study has several strengths. In addition, it focused on a little researched but popular console game.

Yet, the present study had limitations as well. First, our sample was self-selected on social media platforms and may have induced bias in the recruitment process by being conducted in English. Second, our measures relied on self-reports only, which are known to be associated with social desirability, deficits in memory etc. Thirdly, the cross-sectional design used is not suited to test for causal effects statistically. Future research should add to our knowledge by using longitudinal designs, theory-informed SEM, additional objective data, and both quantitative and qualitative methods.

## Conclusion

For some players, casual gaming can become a problematic experience as the high prevalence rate in our sample of ACNH players shows. IGD research needs to diversify its take on game type as well as gamer populations.

## Supplementary Information

Supplementary Table S1.

## Data Availability

The datasets used and/or analysed during the current study available from the corresponding author on reasonable request.
