# Supplementary material for: Differences between recreational gamers and Internet Gaming Disorder candidates in a sample of Animal Crossing: New Horizons players
Source: Sci Rep. 2023 Mar 29;13:5102. doi: 10.1038/s41598-023-32113-6 (PMC10050812; doi:10.1038/s41598-023-32113-6)
Supplement: Supplementary file 1 — Supplementary Table S1. [file 41598_2023_32113_MOESM1_ESM.docx]

| **Supplementary Table S1** | | | |
| --- | --- | --- | --- |
| *Additional Demographic information on the study sample* | | | |
|  |  | *n* | *%* |
| Ethnic identity | Asian | 113 | 3.9 |
|  | Black | 28 | 1 |
|  | Latinx | 147 | 5.1 |
|  | mixed | 173 | 5.9 |
|  | White | 2396 | 82.4 |
|  | other | 52 | 1.8 |
| *Note*. Total sample size was N = 2909. The variable “ethnic identity” was assessed to ensure comparability to the single previous quantitative study on ACNH by Lewis and colleagues [74]. | | | |
